# Intraventricular Administration of Exosomes from Patients with Amyotrophic Lateral Sclerosis Provokes Motor Neuron Disease in Mice

**DOI:** 10.32607/actanaturae.27499

**Published:** 2024

**Authors:** A. V. Stavrovskaya, D. N. Voronkov, A. K. Pavlova, A. S. Olshanskiy, B. V. Belugin, M. V. Ivanova, M. N. Zakharova, S. N. Illarioshkin

**Affiliations:** Research Center of neurology, Ministry of Science and Higher Education of the Russian Federation, Moscow, 125367 Russian Federation; National Research Center for Epidemiology and Microbiology named after the honorary academician N. F. Gamaleya, Moscow, 123098 Russian Federation

**Keywords:** amyotrophic lateral sclerosis, neurodegeneration, motor neurons, exosomes, TDP43

## Abstract

Amyotrophic lateral sclerosis (ALS) is a severe disease of the central nervous system (CNS) characterized by motor neuron damage leading to death from respiratory failure. The neurodegenerative process in ALS is characterized by an accumulation of aberrant proteins (TDP-43, SOD1, etc.) in CNS cells. The trans-synaptic transmission of these proteins via exosomes may be one of the mechanisms through which the pathology progresses. The aim of this work was to study the effect of an intraventricular injection of exosomes obtained from the cerebrospinal fluid (CSF) of ALS patients on the motor activity and CNS pathomorphology of mice. The exosomes were obtained from two ALS patients and a healthy donor. Exosome suspensions at high and low concentrations were injected into the lateral brain ventricles of male BALB/c mice (*n *= 45). Motor activity and physiological parameters were evaluated twice a month; morphological examination of the spinal cord was performed 14 months after the start of the experiment. Nine months after administration of exosomes from the ALS patients, the animals started exhibiting a pathological motor phenotype; i.e., altered locomotion with paresis of hind limbs, coordination impairment, and increasing episodes of immobility. The motor symptoms accelerated after administration of a higher concentration of exosomes. The experimental group showed a significant decrease in motor neuron density in the ventral horns of the spinal cord, a significant increase in the number of microglial cells, and microglia activation. The TDP43 protein in the control animals was localized in the nuclei of motor neurons. TDP43 mislocation with its accumulation in the cytoplasm was observed in the experimental group. Thus, the triggering effect of the exosomal proteins derived from the CSF of ALS patients in the development of a motor neuron pathology in the experimental animals was established. This confirms the pathogenetic role of exosomes in neurodegenerative progression and makes it possible to identify a new target for ALS therapy.

## INTRODUCTION

Amyotrophic lateral sclerosis (ALS) is a severe neurodegenerative disease, which remains incurable today. ALS is characterized by selective degeneration of the upper and lower motor neurons localized in the brain motor cortex and peripheral nuclei, respectively (brainstem and anterior horns of the spinal cord) [1]. Such a localization of the pathological process in ALS leads to progressive neurogenic muscle weakness, which eventually results in the deterioration of such vital functions as breathing and swallowing. This, in turn, inevitably leads to respiratory failure, requiring invasive ventilation and gastrostomy. The disease is characterized by a pronounced clinical heterogeneity, depending on the primary localization of neurodegenerative changes (bulbar and spinal levels), the degree of involvement of the upper and/or lower motor neurons, the progression rate, and the presence of pathogenic mutations.

ALS is an orphan disease; its prevalence is about 5 cases per 100,000 population per year, and the incidence ranges from 2 to 3 cases per 100,000 population per year [2]. The average age of development of the disease’s first symptoms lies in the range of 55 to 65 years; however, in recent decades, there has been a clear trend towards a decrease in the age of the disease onset and an increase in ALS incidence [3]. In most patients, the cause of ALS remains unknown; these cases are classified as sporadic, about 90% of them. Genetically determined (familial) ALS forms associated with causal mutations in various genes account for approximately 10% of all cases [1]. The key molecular drivers in ALS pathogenesis include dysproteostasis, aberrant RNA metabolism, impaired endosomal and vesicular transport, mitochondrial dysfunction, neuroinflammation, etc.; the significance of these elements remains to be clarified [2]. The existence of different genetic forms of ALS makes it possible to design representative cellular and animal models of the disease based on the expression of mutations in the genes *SOD1*, *TARDBP*, *FUS*, etc. in model organisms; transgenic B6SJL-Tg (SOD1–G93A) mice are the most commonly used animals in these experiments [3].

Recent studies hold that extracellular vesicles, mainly exosomes, play a major role in the neurodegenerative progression in the central nervous system (CNS) [4]. Exosomes are encapsulated particles enriched with various molecules, including membrane and cytoplasmic proteins, lipids, and nucleic acids [5]. Exosomes act as effective transport systems and deliver molecular cargo to recipient cells, which makes them one of the most important tools of intercellular communication in both physiological and pathological processes [6]. Exosomes originate from intracellular multivesicular bodies and are 30–150 nm in diameter [7]. During maturation, exosomes are exported to the extracellular space; they can further enter the bloodstream and even cross the blood-brain barrier (BBB) [8]: hence, exosomes can be found in various biological fluids [9, 10]. Many of the protein products of ALSassociated genes are found in exosomes, which enables their transfer between neuronal and glial cells in various brain regions, contributing to the progression of neurodegeneration [11]. These proteins include SOD1, TDP-43, FUS, and proteins with dipeptide repeats characteristic of intracellular inclusions in mutant* C9orf72 *[6]. Braak et al. proposed several hypotheses on neurodegenerative progression in the CNS in ALS [12]. One of the most convincing hypotheses implies the transfer of pathological proteins between adjacent CNS regions via exosome transport. The spread of symptoms to adjacent anatomical regions typical of ALS apparently is clinically a manifestation of the transfer of pathologically aggregated proteins between neighboring cells and within interconnected CNS regions.

The aim of this work was to study the effect of intraventricular administration of an exosome fraction taken from the cerebrospinal fluid (CSF) of patients with sporadic ALS and a healthy donor on the motor activity of model animals and their CNS pathomorphology.

## EXPERIMENTAL

**Obtaining the exosome suspension**

The exosomes used in the study were obtained from two ALS patients. Patient ALS110 is a 48-year-old male with stage 4a cervicothoracic ALS and overall disease duration of 8 months; patient ALS111 is a 67-year-old male with stage 4a cervicothoracic ALS and disease duration of 26 months. The sample material, hereinafter referred to as the control, was obtained from a clinically healthy 57-year-old woman.

The exosomes were isolated from CSF according to the Total Exosome Isolation (from other body fluids) kit instructions (Invitrogen, ref. 4484456). All procedures were performed under aseptic conditions. Prior to isolation, a 0.5 ml CSF aliquot was successively centrifuged at 2,000 *g *and 4°C for 30 min. The cleared supernatant was then centrifuged at 10,000 *g *and 4°C for 30 min. The resulting supernatant was thoroughly mixed with the Total Exosome Isolation reagent, incubated at 2–8°C for 1 h, and the exosomes were pelleted by centrifugation at 10,000 *g *and 2–8°C for 1 h. The resulting pellet was re-suspended in 40 μl of phosphate buffer.

Exosome concentration in the suspension was assessed by evaluating one of its main markers; namely, CD9. The exosome concentration in the purified suspension was 7 × 108; it was designated as high (H). In turn, a suspension with a low (L) concentration of exosomes was obtained by diluting the H suspension 10-fold with phosphate buffer. These two dilutions (H, L) were used for administration to the experimental animals.

**The animals**

The study was performed in male BALB/c mice (*n *= 45) aged 2.5 months (at the beginning of the experiment) and weighing 22–25 g. The animals were obtained from the nursery of the Stolbovaya branch of the Federal State Budgetary Scientific Institution Scientific Center for Biomedical Technologies of the Federal Medical and Biological Agency, Russia. Procedures on the animals were performed in accordance with the requirements of the European Convention for the Protection of Vertebral Animals Used for Experimental and Other Scientific Purposes (CETS No. 170), Order of the Ministry of Health of the Russian Federation No. 119N dated April 1, 2016, On approval of the Principles of good laboratory practice and also guided by the Requirements for working with laboratory rodents and rabbits (National State Standard No. 33216-2014). The animals were kept in standard vivarium conditions with free access to food and water in a 12-hour day/night cycle. Prior to the experiment, the animals had undergone a 14-day quarantine. The study was approved by the Ethics Committee of the Scientific Center of Neurology.

To administer the exosome suspension to mice, the animals were placed in a Lab Standard Stereotaxic Instrument frame (Stoelting, USA) and 2 μl of the suspension were injected bilaterally into the lateral ventricles of the brain through holes drilled in the skull. The administration was performed using the following coordinates from the Mouse Brain Atlas: AP – -0.22; L – 1.0; V – 2.3 [13]. Zoletil-100 (Virbac Sante Animale, France) and Xyla (Interchemie Werken “de Adelaar BV”, Netherlands) were used for anesthesia. A standard Zoletil-100 solution (500 mg in 5 ml) was diluted in saline at a 1 : 4 ratio and injected intramuscularly in an amount of 1.5 mg of the active substance per 25 g of mouse weight. Xylu was diluted in saline at a 1 : 2 ratio and administered intramuscularly in an amount of 0.6 mg per 25 g of mouse weight.

All the animals were divided into five groups of nine mice each: the control and experimental groups, which received drugs at either a high or low dose.

**Physiological study**

The health of the experimental mice was checked twice a week, and changes in motor activity were assessed twice a month. Animal health was examined based on changes in weight, the presence of a porphyrin secretion from the nose and eyes, coat condition, etc. To evaluate the extent of the resulting motor and neurological disorders, the Open Field (OF) and Narrowing Beam (NB) tests were employed.

The OF represented a 40 × 40 × 20 cm box made of polyvinyl chloride (workshops of the Brain Institute of the Scientific Center of Neurology). The mouse was placed in the center, and its motor activity was recorded for 3 min using the ANY-maze Video Tracking software (Stoelting Inc., USA).

The NB setup was composed of two 100-cm long bars superimposed on each other (Open Science, Russia). The width of the upper bar ranged from 0.5 to 2 cm, and the height was 1 cm. The width of the lower bar was 2.5 to 4 cm. The narrow end of the beam had a cage (shelter) with a removable lid and an opening in the frontal panel, through which the animal could get inside. The entire setup was elevated 70 cm above ground. The experimental animal had to traverse the upper bar from the beginning of the path to the shelter. The traversal time and percentage of limb slips onto the lower bar of the total number of steps on the NB were recorded.

Behavioral tests were conducted 11 months after exosome administration. The results are presented in the article.

**Morphological study**

For the morphological study, spinal cord samples were obtained from the ALS111(H) experimental group. The control group consisted of four mice from the same batch as those participating in the experiment. In addition, we used samples from the transgenic ALS model mice (B6SJL-Tg (B6SJL-Tg (SOD1–G93A) line) obtained in our previous study, for comparison [3]. Mice were decapitated, the spine was removed, and the spinal cord was isolated under a binocular microscope. Lumbar regions of the spinal cord were fixed in 4% formalin. After fixation, the samples were immersed in 30% sucrose, placed in an OCT medium, and 12-μm-thick sections were prepared on a Sakura Tissue-Tek cryostat. For immunohistochemical examination, antibodies to the neuronal protein PGP9.5/UCHL1 (ubiquitin carboxyl-terminal hydrolase L1), microglia marker IBA1 (allograft inflammatory factor 1, or ionized calcium binding adaptor molecule 1), and the proteins involved in the pathogenesis of ALS–SOD1 and TDP-43 were used. For antigen retrieval, sections in Tris-EDTA buffer (antigen retrieval solution, pH 9.0, Nordic Biosite) were heated in a steamer for 15 min. The sections were then incubated with primary antibodies. Antibody binding was confirmed using the immunofluorescence method. For this, corresponding secondary goat and donkey antibodies labeled with fluorochromes CF488 and CF555 (Sigma, USA) were used. The reaction was conducted according to the antibody manufacturer’s instructions. In addition, succinate dehydrogenase (SDH) activity in formazan formation [14] was detected in freshly frozen sections of the anterior tibial muscle of two experimental animals after exosome injection and two transgenic SOD1–G93A ALS model mice using the conventional histochemical technique.

The samples were examined on a Nikon Eclipse Ni-U microscope. Neurons were counted using the previously described protocol [15]. The immunofluorescence intensity of IBA1 staining was evaluated using the NIS-Elements software. The assessment was performed in at least 12 L1–L5 sections of the right side of the spinal cord from each animal, and the obtained data were averaged.

The obtained data were processed using the Statistica 12.0 software and one-way analysis of variance (ANOVA) with subsequent post-hoc intragroup comparisons using the Fisher’s criterion for unequal groups, as well as the Mann–Whitney test. The results are presented as the mean and standard error (M ± SEM), indicating the statistical significance of differences between the compared groups for the studied parameters. Differences were considered statistically significant at *p* < 0.05.

## RESULTS

**Fig. 1:**
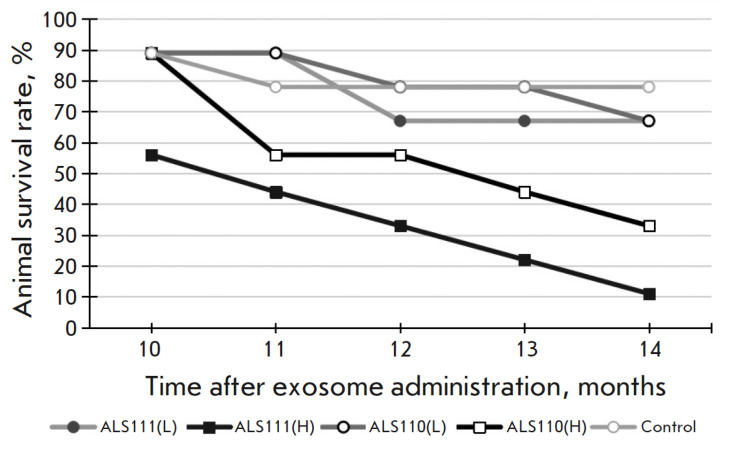
Dynamics of survival of experimental mice in groups

**Fig. 2:**
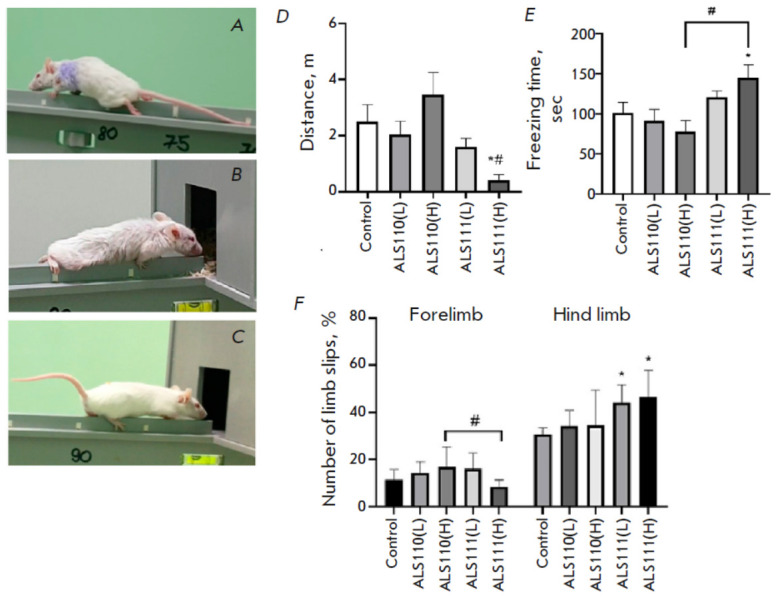
Appearance of G93A (*A*) and ALS111 mice (*B*) after the onset of ALS symptoms. Control animal (*C*); distance traveled (*D*) and immobility time (*E*) during OF testing; number of limb slips from the upper bar (in %) per NB (*E*). * *p* < 0.05 compared to the control group. # *p* < 0.05 compared to the ALS110 (H) group. Data are presented as mean ± SEM

The first signs of motor disorders in individual animals were noted 9 months after exosome administration. By month 10–11, the number of mice with signs of disease had increased (*Fig. 1*), primarily among animals in the ALS111(H) and ALS110(H) groups; i.e., mice receiving a higher drug dose. Examination revealed fur thinning, minor porphyrin discharges from the eyes and nose, and a decrease in body weight (*Fig. 2B*).

Behavioral testing of the animals demonstrated a significant decrease in motor activity, an increase in the period and episodes of immobility in the OF test (*Fig. 2 D,E*), impaired coordination, an increase in the time required to complete the NB test (*Fig. 2F*), and partial paresis of hind limbs. The OF test showed a decrease in the distance traveled (*p *= 0.0276) and an increase in the time of immobility in the ALS111(H) group (*p *= 0.0466) compared to the control group. A decrease in the distance traveled (*p *= 0.0035) and an increase in the immobility time (*p *= 0.0045) were also observed in the ALS111(H) group compared to the ALS110(H) group. The NB test (*Fig. 2F*) showed significant changes in hind limb performance in mice. The number of hind limb slips statistically significantly increased in the ALS111(L) (*p *= 0.0101) and ALS111(H) (*p *= 0.0119) groups compared to the control. A decrease in the number of forelimb slips was also noted in the ALS111(H) group compared to the ALS110(H) group (*p *= 0.04). ALS110(L) and ALS110(H) mice did not show any impairments in the performance of both forelimbs and hind limbs.

Changes observed in mouse appearance, gait, and locomotion after exosome injection differ from the normal age range and are similar to ALS signs in the transgenic B6SJL-Tg (SOD1–G93A) mouse disease model (see *Fig. 2 A–C*).

The animals with the most pronounced motor disorders (dragging up of the hind limbs, impaired gait, and decreased motor activity) were used for histological examination 14 months after the start of the experiment. Performing behavioral tests at this time point proved impossible due to the development of severe neurological disorders by the animals.

**Fig. 3:**
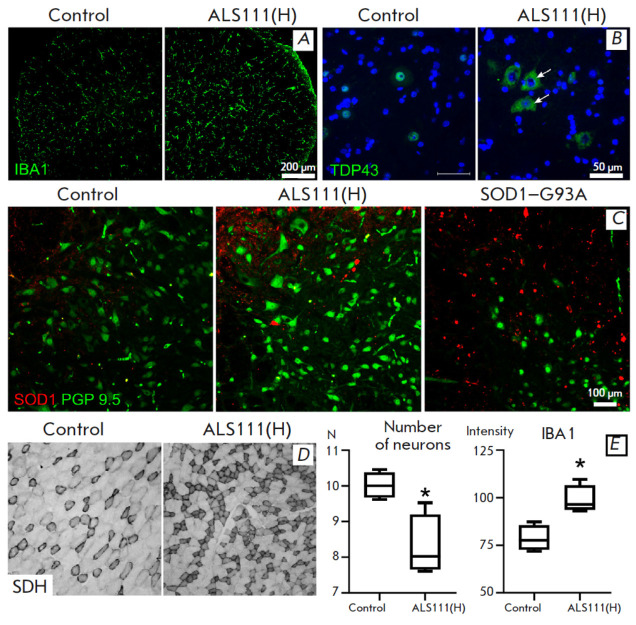
Morphological examination. (*A*) – microglia activation. Microglia marker protein IBA1 staining. Ventral horn of the spinal cord, lumbar region. (*B*) TDP43 localization (green color) in motor neurons. Arrows indicate TDP43 localization in the cytoplasm. Ventral horn of the spinal cord, lumbar section. (*C*) SOD1 accumulation in the spinal cord of experimental animals. SOD1 (red) and PGP9.5 (green) localization. Ventral horn of the spinal cord, lumbar region. (*D*) – increase in the number of SDH-positive fibers. Anterior tibial muscle. (*E*) Changes in motor neurons and neuroglia. Decrease in the number of motor neurons of the ventral horns of the spinal cord (cells in the field of view), increase in the intensity of the staining for the microglia marker protein IBA1. * *p* < 0.05, Mann-Whitney criterion. Data are presented as a median and the interquartile range

Evaluation of the number of motor neurons revealed a significant decrease in their density in the ventral horns of the spinal cord after administration of the high dose of ALS111 compared to the control. In addition, a significant increase in the number of microglial cells and microglia activation in the experimental group, as well as a statistically significant increase in the intensity of staining for the microglial marker IBA1, were noted (*Fig. 3 A,E*).

An analysis of the TDP-43 protein in the neuronal cytoplasm showed a predominant nuclear localization of TDP-43 in the control mice. Protein mislocation, with its accumulation in the cytoplasm, was observed in individual neurons in the experimental group (*Fig. 3B*).

No aggregated SOD1 form was detected in spinal motor neurons in the controls. Individual inclusions of aggregated SOD1 were found in the experimental mice (*Fig. 3C*); however, no pronounced neuronal death was observed. Transgenic SOD1–G93A mice exhibiting multiple SOD1 aggregates and a decreased number of motor neurons in the spinal cord were used as a positive control.

The histochemical response to the SDH activity in the skeletal muscles of the animals revealed a trend towards an increase in the enzyme activity in the experimental group. This process is characteristic of muscle metabolic reprogramming in ALS (*Fig. 3D*); it has been also previously observed in B6SJL-Tg (SOD1–G93A) mice [3].

## DISCUSSION

the progressive death of neurons (with selective vulnerability of individual disease subtypes in specific pathologies) and aggregation of the misfolded proteins that play a key role in the disease [16]. In ALS, the pathological process develops locally with the death of motor neurons and progresses predictably throughout the CNS along certain neuroanatomical pathways [17]. In our out study, we showed that, as early as 9 months after intraventricular administration of exosomes from the CSF of ALS patients, animals began to exhibit a typical motor phenotype: a change in locomotion with paresis of hind limbs, impaired coordination, and an increase in the time and number of episodes of immobility, as demonstrated by physiological studies. This phenotype was similar to that of transgenic animals expressing the G93A mutation in* SOD1 *[3]. It is important to note that the rate of development of motor symptoms depended on the concentration of the injected exosome suspension. This is consistent with the concept of an incubation period required, during which a protein with a prion-like domain (e.g., TDP-43 and SOD1) converts normal protein forms to pathological ones [16, 18]. In the case of a higher concentration of exosomes initiating the neurodegenerative process, this period is reduced.

One of the mechanisms observed in various neurodegenerative diseases is secretion of the native and membrane-associated pathological protein in extracellular vesicles (exosomes) into the extracellular space, followed by its uptake by neighboring cells via receptor- mediated endocytosis or pinocytosis [18, 19, 20]. An alternative mechanism is trans-synaptic transmission through anterograde and/or retrograde transport [21, 22].

One of the main pathomorphological characteristics of ALS is the presence of ubiquitin-positive cytoplasmic inclusions (stress granules) in neurons containing TDP-43 protein aggregates, as observed in autopsy samples from ALS patients [23, 24]. Identification of causal mutations in the *TARDBP *gene encoding TDP-43 confirmed the importance of this protein in the pathogenesis of ALS [25] and frontotemporal dementia (FTD) [26]. The TDP-43 aggregation is observed in neurons in approximately 97% of all ALS cases and almost half of FTD cases [27]. TDP-43 is a highly conserved DNA/RNA-binding protein that executes various functions in the cell, including the regulation of transcription and alternative RNA splicing [28]. TDP-43 consists of four domains: an aminoterminal domain, two RNA recognition motifs, and a carboxyl-terminal domain with prion-like properties [29]. In normal conditions, TDP-43 is located predominantly in the nucleus [30]. In ALS patients, the protein adopts a pathological conformation. Once this protein is captured trans-synaptically by the recipient cell, it interacts with endogenous TDP-43, thus triggering (in the prion-like fashion) aggregation of intrinsic TDP-43 and thereby spreading the pathology to other CNS structures [31, 32]. The CSF and CNS tissue from ALS and FTD patients has been shown to cause TDP-43 aggregation and induce TDP-43 proteinopathy in both cell cultures and *in vivo *[32, 33, 34]. In such diseases as ALS or FTD, TDP-43 mislocation and an increase in its cytoplasmic level are noted in the cell, which results in the formation of protein inclusions in the cytoplasm and impairment of its functions in the cell nucleus [35]. In our study, we used spinal cord samples from mice with the most pronounced motor disorders (14 months after the start of the experiment) for immunohistochemical analysis, which demonstrated TDP-43 mislocation, with predominant accumulation in the neuron cytoplasm. These results are consistent with the data obtained by other researchers.

There is data on the intercellular transport of TDP-43 aggregates via exosomes [36]. Exosomal secretion of such pathological proteins as β-amyloid, Tau, the prion protein, and α-synuclein was also reported in other neurodegenerative diseases [16, 20]. Exosomal transport of TDP-43 plays an important role in ALS pathogenesis, since significantly higher levels of exosomal TDP-43 are detected in the brain and CSF biopsy samples from ALS patients compared to controls [33, 37].

In addition to transmission between neurons, the spread of pathological proteins between neurons and glia (astrocytes, microglia, and/or oligodendrocytes) has also been reported [20]. For instance, the Tau protein can enter astrocytes [38] and microglia, which play a key role in the spread of pathological Tau protein via exosome transport [38, 39]. In our study, staining of spinal cord samples from the experimental animals for the microglial marker IBA1 revealed an increase in the number of microglial cells and their activation, which indicates a direct involvement of innate immunity in the molecular mechanisms of motor neuron death. The inflammatory response occurring in the pathology has some beneficial effects, restoring tissue integrity and homeostasis; however, chronic neuroinflammation depletes the regenerative potential of microglia [40]. Microglia is activated via inflammasomes, which are high-molecular complexes in the cytosol of immune cells that mediate the activation of pro-inflammatory caspases [41]. A crucial intracellular factor inflammasomes respond to in ALS is the accumulation of toxic aggregates of the TDP-43, SOD1, and other proteins that cause neuroinflammation in neurons [42]. The inflammasome activation cascade initiates the release of interleukins (IL)-1β and IL-18 and causes pyroptosis. Pyroptosis is programmed cell death mediated by gasdermin D and the influx of sodium ions and water, which lead to cell swelling with membrane rupture and the release of the cytosol content into the extracellular space, resulting in the spread of pathological proteins in CNS cells [43].

In contrast to sporadic ALS forms, which are characterized by the presence of TDP-43 as the main component of intracellular inclusions [24], familial disease forms with a verified mutation in *SOD1 *are characterized by predominant deposition of the mutant SOD1 protein [44]. It is important to note that cellular aggregates of wild-type SOD1 are also detected in some other familial ALS cases and individual cases of sporadic forms lacking *SOD1 *mutations [45]. This might explain the aggregated SOD1 deposits we found in the experimental animals lacking the *SOD1* mutation. In addition, the low amount of deposits explains the relative preservation of motor neurons. At the same time, analysis of the positive control (SOD1– G93A transgenic mice) revealed multiple SOD1 aggregates and a decreased number of motor neurons, which die as a result of the toxic effect of SOD1 on the cell through the gain-of-function mechanism.

In recent years, several innovative approaches to ALS treatment using exosomes and extracellular vesicles have been proposed [46]. Most of these methods involve the use of exosomes for a targeted delivery of various neurotrophic factors and microRNAs through the BBB in order to inhibit the motor neuron death. Considering the fact that, in the ALS pathogenesis, exosomes presumably mediate one of the main mechanisms of pathology progression in the CNS, which is also shown in the present work, we can contemplate the possibility of modulating the neurodegenerative process by inhibiting exosome transport at its various stages. One such promising method aimed at inhibiting the spread of the neurodegenerative process by exosomes is immune blocking of exosome fusion with the motor neuron membrane using anti-CD63 antibodies and, presumably, other key exosome markers [47]. In addition to such an effect on exosomes, an important issue in inhibiting the exosomic pathway in ALS remains the development of drugs that selectively block the transfer of proteins with an altered conformation and prion-like properties.

Thus, in this study, we demonstrated the triggering effect of exosomal proteins from the CSF of ALS patients in the development of motor neuron death in experimental animals. The presented data confirm the pathogenetic role of exosomes in the spread of the neurodegenerative process in the disease and open up a possibility for identifying new targets for ALS therapy.

## Abbreviations

ALS: amyotrophic lateral sclerosis
FTD: frontotemporal dementia
OF: open field test
NB: narrowing beam test
SDH: succinate dehydrogenase
CNS: central nervous system
CSF: cerebrospinal fluid
IBA1: allograft inflammatory factor 1, or ionized calcium binding adaptor molecule 1
IL: interleukin
PGP9.5/UCHL1: protein gene product 9.5/ubiquitin carboxyl-terminal hydrolase L1
